# Curcumin supplementation accelerates high-altitude acclimatization, prevents polycythemia and modulates gut microbiota in male Han population: a randomized controlled trial

**DOI:** 10.3389/fnut.2025.1572376

**Published:** 2025-07-08

**Authors:** Jilei Hu, Hongmei Lang, Die Fan, Ting Wen, Jiaojiao Shi, Chunxiu Xiao, Yunming Li, Chao Kang, Peijie Shi, Lirong Shen, Ning Lin

**Affiliations:** ^1^Department of Clinical Nutrition, The General Hospital of Western Theater Command, Chengdu, China; ^2^Department of General Medicine, Chengdu Second People’s Hospital, Chengdu, China; ^3^Department of Physical Examination Center, The General Hospital of Western Theater Command, Chengdu, China; ^4^Department of Data and Intelligence, The General Hospital of Western Theater Command, Chengdu, China; ^5^College of Medicine, Southwest Jiaotong University, Chengdu, China; ^6^Department of Food Science and Nutrition, Zhejiang University, Hangzhou, China; ^7^College of Public Health, Chengdu Medical College, Chengdu, China

**Keywords:** curcumin, gut microbiota, hemoglobin, mountain sickness, high altitude polycythemia, high altitude acclimatization

## Abstract

**Background:**

Previous evidence showed that curcumin enhanced the oxygen supply efficiency of hemoglobin and alleviated acute plateau hypoxia injury in animal models. However, its efficacy on human beings is not yet verified. This study aimed to assess the effects of curcumin supplementation on hypoxia injury and gut microbiota in the male Han population.

**Methods:**

In this 7-week single-blinded randomized trial, 102 male Han population urgently entered the 3,000 meters altitude from the plain and received 812 mg curcumin or placebo per day for 1 week on the plain and 6 weeks on the plateau. Biochemical parameters were assessed and physical examination was carried out at the baseline (T0), and the end of the 1st (T_1_) and 7th week (T_3_) of intervention. The score of acute mountain sickness (AMS) was evaluated in the 2nd week after entering the plateau (T_2_) and T_3_. Intestine microbial composition was analyzed by metagenomic sequencing.

**Results:**

After a 1-week intervention on the plain, curcumin significantly increased red blood cells (RBC), hematocrit (HCT), and hemoglobin in treatment group as compared to placebo group (*p* < 0.05). However, curcumin significantly reduced the levels of HCT and hemoglobin compared to that in the placebo group after the 6-week intervention on the plateau (*p* < 0.05). Furthermore, the score of AMS in the curcumin group were lower than those in the placebo group at T_3_, although with no significant difference. Gut microbiota analysis indicated that curcumin significantly increased the abundance of butyrate-producing bacteria *Roseburia*, *Lachnospira,* and *Sellimonas* while decreasing the abundance of *Alistipes* and *Escherichia* at high-altitude environments. In addition, a higher relative abundance of *Bifidobacterium* was observed in the curcumin group on the plateau.

**Conclusion:**

Curcumin exhibited different regulation of hemoglobin in low- and high-altitude environments. On the plain, curcumin supplementation elevated the RBC and hemoglobin, which is favorable for reducing the incidence of AMS at the early stage of entering the plateau. On the plateau, curcumin suppressed excessive increase of HCT and hemoglobin by modulating the abundance of butyrate-producing bacteria to avoid the occurrence of high-altitude polycythemia.

**Clinical trial registration:**

https://www.chictr.org.cn/, identifier: ChiCTR220005965.

## 1 Introduction

When people from the plains rapidly enter the plateau, a series of physiological damage occurs in the body with mild symptoms manifesting as headache, nausea, diarrhea, bloating, and sleep disturbance. Severe complications develop into high-altitude pulmonary edema (HAPE), high-altitude cerebral edema (HACE), and high-altitude polycythemia (HAPC) (1, 2). The acute response stage occurs within 7 days of arriving at high altitudes. It is often accompanied by acute mountain sickness (AMS), characterized by a series of pathological changes such as headache, dizziness, and gastrointestinal upset which can be life-threatening in severe cases (3). Acclimatization to high altitudes often occurs after 30 days of entering the plateau, however, prolonged exposure to hypoxia may lead to chronic mountain sickness (CMS). The most prevalent CMS is HAPC reflected by excessively increased hemoglobin (females, hemoglobin 190 g/L or more; males, 210 g/L or more) in patients living above an altitude of 2,500 m (4, 5). Secondary thrombosis, bleeding, extensive organ damage, and sleep disturbances lead by HAPC pose a health risk to high-altitude populations (6).

Hemoglobin is a key protein that carries and releases oxygen in the body and plays an important role in maintaining the normal physiological activities of the body. During the pre-plateau and early plateau periods, an appropriate increase in hemoglobin helps to maintain body homeostasis and improves tissue oxygenation to some extent for altitude acclimatization (7–9). However, a chronic and excessive increase in hemoglobin leads to higher blood viscosity and impaired blood microcirculation, ultimately generating severe hypoxia and irreversible lesions of cardiorespiratory function (10). Tibetans who have inhabited locations at high altitudes for generations present strong adaptability (11), with a lower hemoglobin than the Han population living at the same high altitude, have developed a unique resilience to HAPC by the higher oxygen affinity of hemoglobin (12, 13). Therefore, simply increasing hemoglobin quantity does not alleviate plateau hypoxic injury, while elevating the affinity of hemoglobin for oxygen is more effective (14).

The oxygen homeostasis in the gut is one of the key factors in maintaining the assembly of the gut microbial community. As the altitude increased, the abundance of obligate anaerobes tended to elevate while that of aerobic bacteria decreased significantly (15). Hypoxia in both humans and animals can affect intestinal microbiota composition (16). Intestinal microbiota-mediated energy generation and blood pressure regulation are two major beneficial physiological effects of gut microbiota in high-altitude environments (17–19). Some native high-altitude animals and humans share several genes and hold a greater relative abundance of intestinal bacteria, such as *Lachnospiraceae* and *Ruminococcaceae*, which have been known to produce short-chain fatty acids (SCFA), minimize energy loss, and enhance adaption to high-altitude environments (20–23). The Mendel randomization analysis based on genome-wide association analysis data has shown that erythrocyte counts were negatively correlated with *Bilophila*, *Prevotella* 9, *Eubacterium oxidoreductase*, *Lachnospira*, and *Ruminococcaceae UCG005* in humans at high altitudes (24).

Prior acclimatization for at least 7 days before entering the plateau can effectively minimize the acclimatization time for people’s rapid translocation to high altitudes. Some findings suggest that pre-administration of traditional Chinese medicines such as *Rhodiola rosea* effectively ameliorates acute plateau hypoxia (25). Curcumin, isolated from the plant turmeric (*Curcuma longa* L.), is the active polyphenol in turmeric, commonly used to prepare spices and traditional medicine (26). Accumulating evidence from *in vitro* and *in vivo* experiments has shown that curcumin significantly protects the respiratory, nervous, cardiovascular, and skeletal muscle systems in a hypoxia environment (27, 28). *In vitro* studies found that curcumin could bind to the heme region on the *α*-subunit of hemoglobin, increasing the structural stability of oxyhemoglobin and enhancing its oxygen-carrying capacity (29). Furthermore, animal studies reported curcumin could effectively improve the oxygen supply efficiency of hemoglobin in rats with acute high-altitude hypoxia, inhibit the compensatory increase in red blood cells (RBC), reduce organ damage, and effectively alleviate acute high-altitude hypoxia injury (30).

Curcumin preferentially accumulates in the gastrointestinal system after being administered orally or intraperitoneally, suggesting that curcumin may directly impact gut microbiota (31, 32). The poor aqueous solubility, bioavailability, and pharmacokinetic profiles of curcumin limits its therapeutic usage (33). Numerous efforts have been made by formulating curcumin with adjuvants, nanoparticles, liposomes, and micelles to increase its oral bioavailability, which was enhanced via nanotechnology in the form of a water-soluble turmeric preparation PURCUMIN^®^ (34). Animal experiments showed that the bioavailability of PURCUMIN^®^ was increased by 18.46 times than that of regular curcumin (35, 36), meanwhile, its light stability was also enhanced. Despite promising animal data, proof-of-concept studies in humans are lacking. Given the link between gut microbiota, high-altitude acclimatization, and hemoglobin, we conducted a randomized controlled trial to examine the effects of curcumin on hypoxia injury via gut microbiota modulating in Han population rapidly entering the plateau, trying to understand its potential efficacy in high-altitude acclimation.

## 2 Participants and methods

### 2.1 Materials

Curcumin standard was purchased from the China Institute for Food and Drug Control (Beijing, China). PURCUMIN^®^ gummies and placebo were prepared by Henan Zhongda Hengyuan Biotechnology Stock Co., Ltd. (Luohe, China). Placebo dyed with gardenia yellow have similar colors to curcumin gummies. The mean weight of every gummy was 5 g. Test results of high-performance liquid chromatography according to the reported method (37) showed that curcumin gummies contained curcumin 1.16 g/100 g (Supplementary material 1).

### 2.2 Participant enrollment and informed consent assignment

In this study, we only recruited young male Han subjects from Sichuan Province to avoid any influence of sex differences. The major inclusion criteria were healthy young (18 to 45 years) males from Han region, born and previously lived in low-altitude areas (< 500 m), with routine physical activity habits (1–2 h moderate training intensity). Individuals with acute or chronic organ diseases, such as acute or chronic respiratory diseases, primary cardio cerebral vascular disease, asthma, digestive disease, food allergy, or skin diseases, were excluded. To verify the self-reported healthy condition, a brief physical examination was given to all the participants to confirm the results. Without a similar pilot trial or data to refer to, we assume a 10% increase in hemoglobin in the intervention group. Using a power of 80% and a type one error rate of 5%, the number of participants per group was estimated as 35. To avoid a high drop-out rate influencing the result, 40% was added to the sample calculation, with 49 subjects in each group. According to our protocol, we recruited 140 young male subjects and finally assigned subjects signed informed consent to the placebo group (*N* = 51) and the curcumin group (*N* = 51) by computer-generated randomization. The study protocol was reviewed and approved by the ethics committee of The General Hospital of the Western Theater Command (No. 2021EC3-45) and adhered to the principles of the Declaration of Helsinki. The study was registered at the Chinese Clinical Trial Registry (No. ChiCTR2200059652).

### 2.3 Study design

This was a parallel single-blinded study. Subjects were randomized into the placebo and the curcumin groups according to the randomization list generated by SPSS 22.0. The participants in the curcumin group received 7 curcumin gummies in the morning and evening every day (14 gummies per day, total curcumin 812 mg per day) for 1 week on the plain and 6 weeks on the plateau, and the subjects in the placebo group took equivalent gardenia yellow gummies.

**Figure 1:**
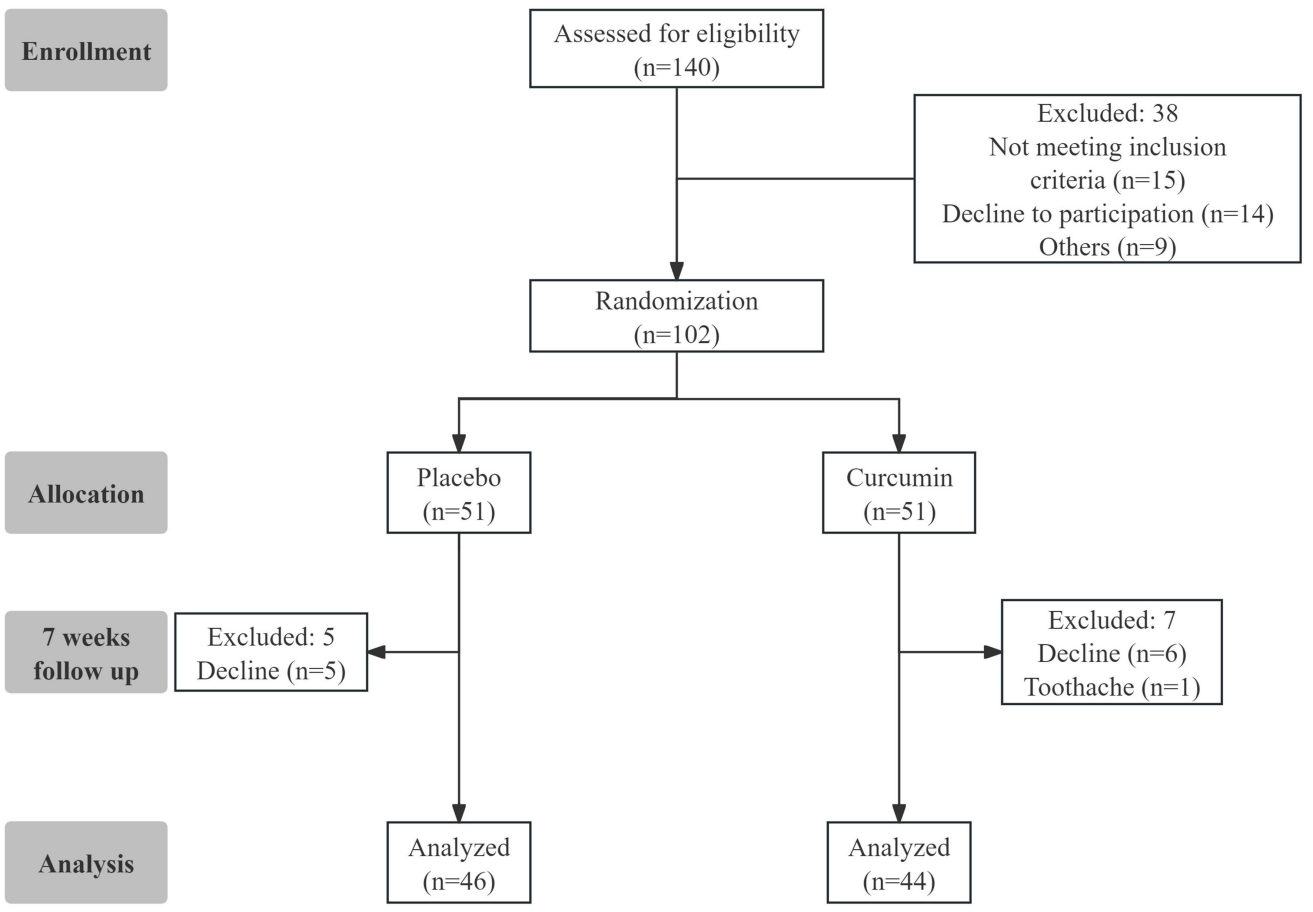
Diagram of the study design.

The flowchart of the study design is shown in Figure 1. A total of 102 eligible participants were randomly assigned to the two groups of Placebo and Curcumin. Twelve subjects were lost in the follow-up from the study because of gummy discontinuation due to personal reasons. Ninety subjects (*n* = 44 in the curcumin group and *n* = 46 in the placebo group) completed the trial. Compliance with the study protocol was good for those who accomplished the study. No serious side effects related to curcumin consumption were observed or reported during the study.

### 2.4 Blood sample collection and test

Blood samples were collected and physical examination was carried out 3 times at baseline (T_0_), 1st week on the plain (T_1_), and 6th week after entering plateau (T_3_). AMS scoring was evaluated at the end of 2nd week after entering the plateau (T_2_) and at T_3_. Serum was obtained after blood centrifugation at 2000 rpm for 10 min at 4°C. All samples were aliquoted and immediately stored at −80°C. Biochemical parameters were assessed by department of clinical laboratory of the General Hospital of Western Theater Command. Complete blood count including hemoglobin, RBC, mean corpuscular hemoglobin concentration (MCHC) and hematocrit (HCT) were measured by automated hematology pipeline system (Mindray Medical International, CAL8000, China). The activity of alkaline phosphatase (ALP) and creatine kinase (CK), and concentration of urea nitrogen (UN), creatinine (CREA), and uric acid (UA) were test by automatic biochemical analyzer (Beckman Coulter, AU5800, United States). Abdominal ultrasound was used for liver and kidney examinations by color Doppler ultrasound diagnostic system (Mindray Medical International, M55, China).

### 2.5 Diagnostic criteria and assessment of AMS

AMS is defined as the 2018 Lake Louise AMS score total of three or more points from the four rated symptoms including at least one point from headache in the setting of a recent ascent or gain in altitude (3). The four rated symptoms of AMS including headache, gastrointestinal symptoms, fatigue and/or weakness, and dizziness/light-headedness. Each symptom is scored on a 0–3 scale (0 = absent; 1 = mild; 2 = moderate; 3 = severe/incapacitating). Furthermore, 3–5 points were classified as mild AMS, 6–9 points as moderate AMS, and 10–12 points as severe AMS.

### 2.6 Gut microbiota analyses

Stool samples were collected from participants at baseline (T_0_) and 6th week after entering plateau (T_3_) for sequencing. These samples were stored by flash freezing at −80°C to preserve the integrity of the microbial DNA. Genomic DNA was extracted from fecal samples and concentration of DNA was measured using Qubit® dsDNA Assay Kit in Qubit® 2.0 Flurometer (Life Technologies, CA, United States). Sequencing libraries were generated using NEBNext® Ultra™ DNA Library Prep Kit for Illumina (NEB, United States). After cluster generation, the library preparations were sequenced on an Illumina Novaseq 6,000 platform and paired-end reads were generated (Beijing Novogene Co. Ltd). The clustering and annotation of OTU were performed to analyze the diversity index, the community structure, and species abundance at each taxonomic level.

### 2.7 Statistical analysis

All statistical analyses were two-tailed and performed using SPSS V22.0 (SPSS Inc., Chicago, IL, United States). Statistical significance was defined as *p* < 0.05. Continuous statistical variables were expressed as mean ± SEM. The changes from T_0_ to T_1_ were calculated as the values after 1 week of intervention deducted values at baseline, and the changes from T_1_ to T_3_ were calculated as the values after 7 weeks of intervention deducted values after 1 week of intervention. For quantitative data, the Shapiro–Wilk test was applied to determine the normality of the variable distribution. For normally distributed data, differences between the groups were analyzed by independent samples *t*-test, and differences within groups were evaluated by paired sample *t*-test. Repeated measures ANOVA was used to analyze the changes in variables over time. For not normally distributed data, differences between groups were compared by Wilcoxon rank sum test, and differences within groups were compared by Wilcoxon paired signed-rank test.

## 3 Results

### 3.1 Demographic data and baseline clinical biochemistry of study participants

The baseline characteristics of all 102 eligible participants are shown in Table 1. At baseline, both groups of participants were comparable in age, height, weight, BMI, population sources, residential area, educational background, smoking, and nutritional supplementation intake. No subject had consumed nutritional supplementation related to the promotion of high-altitude acclimatization. No significant differences in hemoglobin, MCHC, SaO_2_, SBP, DBP, and HR were observed between the two groups at baseline.

**Table 1:** Demographic data and baseline clinical biochemistry of study participants.

Variables	Curcumin (*n* = 51)	Placebo (*n* = 51)	*p* (between groups)
Age (y)	20.75 ± 1.78	20.31 ± 1.64	0.206
Height (cm)	173.66 ± 5.81	175.68 ± 5.60	0.077
Weight (kg)	70.61 ± 8.63	71.76 ± 6.93	0.459
BMI (kg/m^2^)	23.37 ± 2.09	23.25 ± 1.89	0.762
Population sources (n, %)			0.674
City	18 (35.29)	16 (31.37)	
Rural	33 (64.71)	35 (68.63)	
Residential Area (n, %)			0.541
South	18 (35.29)	21 (41.18)	
North	33 (64.71)	30 (58.82)	
Educational background (n, %)			0.088
High School and above	20 (39.22)	12 (23.53)	
Others	31 (60.78)	39 (76.47)	
Smoking, n (%)	30 (58.90)	26 (50.00)	0.426
Nutritional supplementation intake, n (%)	1 (1.96)	0 (0.00)	1.000
RBC (×10^12^/L)	4.89 ± 0.04	4.77 ± 0.04	0.060
Hemoglobin (g/L)	147.63 ± 1.04	147.00 ± 1.20	0.684
MCHC (g/L)	329.30 ± 1.03	329.12 ± 1.02	0.904
HCT (g/L)	44.95 ± 0.29	44.69 ± 0.35	0.568
SaO_2_ (%)	97.67 ± 0.16	97.65 ± 0.15	0.928
HR (bpm)	82.24 ± 1.39	78.88 ± 1.47	0.101
SBP (mm Hg)	119.82 ± 1.51	118.04 ± 1.31	0.374
DBP (mm Hg)	65.98 ± 0.81	65.41 ± 1.07	0.673

Values are presented as mean ± SEM or number (%). *p* (between groups), the differences between groups were compared by independent sample *t*-test or chi-square test. BMI, body mass index; RBC, red blood cell counts; MCHC, mean corpuscular hemoglobin concentration; HCT, hematocrit; SaO_2_, transcutaneous oxygen saturation; HR, heart rate; SBP, systolic blood pressure; DBP, diastolic blood pressure.

### 3.2 Effect of curcumin supplementation on hepatic and renal functions

Activity of ALP and CK, and concentration of UN, CREA, and UA in both groups at T_0_ were comparable (Table 2). There was no significant difference between groups regarding activity of ALP and CK, and concentration of UN and UA at T_3_. However, the level of CREA in the curcumin group significantly increased at T_3_ compared with that in the placebo group (*p* < 0.05). At the end of the trial, abdominal ultrasound results revealed that five subjects had intrahepatic calcified foci (11.36%), two subjects suffered from gallbladder polyp (4.55%), and five subjects were found to have kidney crystals (11.36%) in the curcumin group. In comparison, four subjects had intrahepatic calcified foci (8.7%), two subjects contracted mildly fatty liver (4.35%), one subject had hepatic hemangioma (2.17%), one subject suffered from gallbladder polyp (2.17%) and four subjects were found to have kidney crystals (8.7%) in the placebo group. No significant differences in abdominal ultrasound results were observed between the two groups.

**Table 2:** Effect of curcumin supplementation on hepatic and renal functions.

Index	Curcumin (*n* = 44)	Placebo (*n* = 46)	*p* (between groups)
ALP (IU/L)			
T_0_	86.51 ± 2.93	92.20 ± 3.04	0.181
T_3_	113.87 ± 3.13	118.96 ± 4.04	0.325
CK (IU/L)			
T_0_	213.25 ± 11.36	229.60 ± 14.02	0.370
T_3_	262.24 ± 13.12	261.46 ± 16.40	0.971
UN (mmol/L)			
T_0_	5.52 ± 0.19	5.57 ± 0.18	0.851
T_3_	6.52 ± 0.21	6.13 ± 0.17	0.157
CREA (μmol/L)			
T_0_	82.98 ± 1.36	83.07 ± 1.18	0.961
T_3_	100.82 ± 2.02	94.91 ± 1.26	**0.014**
UA (μmol/L)			
T_0_	435.41 ± 12.54	437.11 ± 12.64	0.924
T_3_	477.55 ± 15.20	449.96 ± 11.57	0.150
Intrahepatic calcified foci	5 (11.36%)	4 (8.70%)	0.673
Mildly fatty liver	0 (0.00)	2 (4.35%)	0.495
Hepatic hemangioma	0 (0.00)	1 (2.17%)	1.000
Gallbladder polyp	2 (4.55%)	1 (2.17%)	0.612
Kidney crystals	5 (11.36%)	4 (8.70%)	0.737

Values are presented as mean ± SEM or counts (%). *p* (between groups), the differences between groups were compared by independent sample *t*-test for continuous variables; the differences between groups were compared by chi-square test or Fisher’s exact probability test for categorical variables. ALP, alkaline phosphatase; CK, creatine kinase; UN, urea nitrogen; CREA, creatinine; UA, uric acid. *p*-values less than 0.05 are bolded, indicating that these results are statistically significant.

### 3.3 Effect of curcumin supplementation on high altitude acclimatization

The blood pressure levels, oxygen saturation, hemoglobin, and HR in the curcumin and placebo groups were comparable at baseline. As shown in Table 3, significant differences in the hemoglobin, SBP, and DBP between curcumin and the placebo groups at T_3_ (*p* < 0.01). Curcumin significantly increased the hemoglobin, RBC, and HCT at T_1_ when compared with that of the placebo group (*p* < 0.05), while the hemoglobin and HCT in the curcumin group were lower than those in the placebo group at T_3_ (*p* < 0.05). The changes in hemoglobin, RBC, and HCT from T_0_ to T_1_ in the curcumin group were significantly higher than those in the placebo group (*p* < 0.01, *p* < 0.05), but the changes in the above three indexes from T_1_ to T_3_ in the curcumin group were significantly lower than in the placebo group (*p* < 0.01). The MCHC significantly increased in both groups at the end of the study in comparison with the baseline value (*p* < 0.01).

**Table 3:** Effect of curcumin supplementation on hematology and blood pressure during high altitude acclimatization.

Index	Curcumin (*n* = 44)	Placebo (*n* = 46)	*p* (between groups)	*p* (time×group)
Hemoglobin (g/L)				**< 0.001**
T_0_	147.09 ± 1.21	146.70 ± 1.81	0.816	
T_1_	160.18 ± 1.35^**^	155.57 ± 1.32^**^	**0.016**	
T_3_	170.95 ± 1.72^**^	176.07 ± 1.69^**^	**0.037**	
T_1_-T_0_	13.09 ± 0.96	8.87 ± 1.11	**0.005**	
T_3_-T_1_	10.77 ± 0.83	20.50 ± 1.59	**< 0.001**	
RBC (×10^12^/L)				**< 0.001**
T_0_	4.87 ± 0.04	4.76 ± 0.04	0.051	
T_1_	5.41 ± 0.06	5.12 ± 0.05	**< 0.001**	
T_3_	5.67 ± 0.07	5.67 ± 0.0.07	0.971	
T_1_-T_0_	0.54 ± 0.05	0.37 ± 0.04	**0.004**	
T_3_-T_1_	0.26 ± 0.03	0.55 ± 0.05	**< 0.001**	
MCHC (g/L)				0.531
T_0_	328.84 ± 1.09	329.20 ± 1.06	0.816	
T_1_	328.64 ± 0.78	327.47 ± 0.76	0.288	
T_3_	358.95 ± 1.04^**^	358.83 ± 1.02^**^	0.930	
T_1_-T_0_	−0.20 ± 1.06	−1.72 ± 1.05	0.313	
T_3_-T_1_	30.31 ± 0.80	31.35 ± 0.76	0.352	
HCT (g/L)				**< 0.001**
T_0_	44.86 ± 0.35	44.59 ± 0.35	0.596	
T_1_	48.75 ± 0.38	47.50 ± 0.37	**0.022**	
T_3_	47.62 ± 0.41	49.04 ± 0.41	**0.016**	
T_1_-T_0_	3.89 ± 0.29	2.91 ± 0.33	**0.030**	
T_3_-T_1_	−1.13 ± 1.62	1.54 ± 0.40	**< 0.001**	
SaO_2_ (%)				0.083
T_0_	97.66 ± 0.17	97.70 ± 0.16	0.875	
T_1_	98.27 ± 0.12	98.13 ± 0.11	0.382	
T_3_	94.75 ± 0.27	94.65 ± 0.26	0.795	
T_1_-T_0_	0.61 ± 0.19	0.43 ± 0.20	0.517	
T_3_-T_1_	−3.52 ± 0.24	−3.48 ± 0.27	0.903	
SBP (mm Hg)				**0.001**
T_0_	119.23 ± 1.55	117.74 ± 1.39	0.474	
T_1_	111.66 ± 1.27^**^	117.35 ± 1.63	**0.007**	
T_3_	111.75 ± 1.45^**^	114.63 ± 1.51	0.173	
T_1_-T_0_	−7.57 ± 1.70	−0.39 ± 1.73	**0.004**	
T_3_-T_1_	0.09 ± 1.69	−2.72 ± 1.51	0.217	
DBP (mm Hg)				**0.007**
T_0_	65.25 ± 0.83	65.65 ± 1.16	0.417	
T_1_	65.07 ± 0.97	67.35 ± 1.43	0.196	
T_3_	67.98 ± 1.06	72.70 ± 1.25^**^	**0.005**	
T_1_-T_0_	−0.18 ± 0.99	1.70 ± 1.15	0.220	
T_3_-T_1_	2.91 ± 0.97	5.35 ± 1.50	0.180	

Values are presented as mean ± SEM. *p* (time×group), an interaction effect between intervention and time was analyzed by repeated measures ANOVA. *p* (between groups), the differences between groups were compared by independent sample *t*-test. ^*^p < 0.05, ^**^p < 0.01, the differences between baseline and post-intervention data within groups were compared by paired sample *t*-test. *p*-values less than 0.05 are bolded, indicating that these results are statistically significant.

Curcumin intervention significantly decreased the SBP at T_1_ when compared with placebo (*p* < 0.01), while no significant differences were observed at T_3_ between the two groups. The SBP decreased from T_0_ to T_1_ in both groups, but the curcumin group experienced a greater decrease than the placebo group (*p* < 0.01). No significant differences in DBP were observed at T_1_ between the two groups, however, the DBP in the curcumin group was significantly lower than that in the placebo group at T_3_ (*p* < 0.01). The SBP decreased significantly in the curcumin group at T_0_ and T_3_ in comparison with the baseline level (*p* < 0.01). However, the DBP was increased significantly in the placebo group at the end of the intervention when compared with the baseline value (*p* < 0.01).

### 3.4 Effect of curcumin supplementation on AMS

As shown in Table 4, no significant differences were observed in the scores of AMS or AMS symptoms between curcumin and placebo groups at T_2_ or T_3_. The scores of gastrointestinal symptoms, fatigue and/or weakness, and AMS in the curcumin group were lower than those in the placebo group at T_3_, though the differences were not significant (*p* > 0.05). Notably, the placebo group exhibited significantly higher scores for headaches, fatigue and/or weakness, gastrointestinal symptoms, and AMS at T_3_ compared with T_2_ (*p* < 0.01). Similarly, the scores of headaches and AMS increased significantly in the curcumin group at T_3_ compared with T_2_ (*p* < 0.01).

**Table 4:** Effect of curcumin supplementation on AMS and AMS symptoms of subjects at T_2_ and T_3_.

Symptom	T_2_			T_3_		
	Curcumin (*n* = 44)	Placebo (*n* = 46)	*p* (between groups)	Curcumin (*n* = 44)	Placebo (*n* = 46)	*p* (between groups)
Headaches	0.34 ± 0.07	0.35 ± 0.07	0.945	0.68 ± 0.08^**^	0.63 ± 0.08^**^	0.632
Gastrointestinal symptoms	0.20 ± 0.07	0.13 ± 0.05	0.480	0.20 ± 0.06	0.28 ± 0.07^**^	0.392
Fatigue and/or weakness	0.50 ± 0.08	0.39 ± 0.07	0.745	0.86 ± 0.06	1.00 ± 0.08^**^	0.182
Dizziness/light-headedness	0.70 ± 0.08	0.74 ± 0.08	0.302	0.57 ± 0.08	0.43 ± 0.07	0.208
AMS	1.75 ± 0.20	1.61 ± 0.17	0.841	2.32 ± 0.17^**^	2.35 ± 0.20^**^	0.881

Values are presented as mean ± SEM. *p* (between groups), the differences between groups were compared by Wilcoxon rank sum test. ^*^p < 0.05, ^**^p < 0.01, the differences between T_2_ and T_3_ within groups were compared by Wilcoxon paired signed-rank test.

### 3.5 Effect of curcumin supplementation on the diversity of gut microbiota

**Figure 2:**
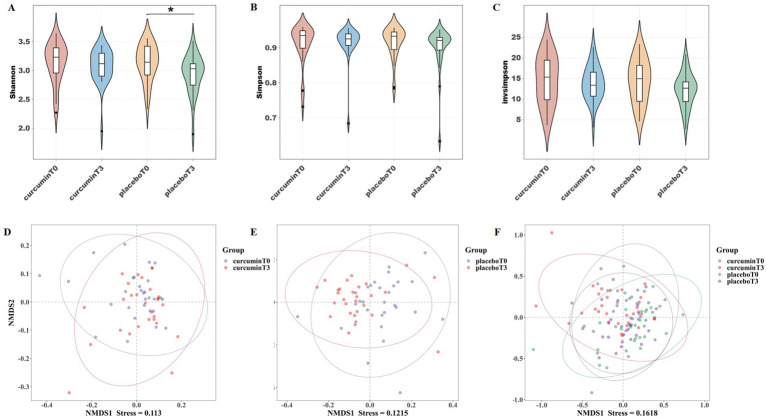
*α*-diversity indexes of **(A)** Shannon, **(B)** Simpson, and **(C)** Invsimpson; and *β*-diversity analysis of NMDS **(D–F)** observed at T_0_ and T_3_ in the placebo and curcumin groups. ^*^p < 0.05, ^**^p < 0.01, and ^***^p < 0.001.

The Shannon index of the placebo group was significantly higher on plains than on plateaus (*p* < 0.05), however, no significant difference between the two groups at two time points was observed in the Shannon, Simpson, and Invsimpson indexes (Figures 2A–C). Non-metric multidimensional scaling (NMDS) analysis showed a stress value of 0.113, 0.122, and 0.162, with a stress value of less than 0.2 indicating reliable data results (Figures 2D–F). After hypobaric hypoxia exposure for 6 weeks, the composition of the gut microbiota was changed in the placebo group. After the treatment of curcumin, there was a clear separation of microbial structure. NMDS analysis suggested noticeable differences in the composition of the microbial communities among the four groups.

### 3.6 Effect of curcumin supplementation on the relative abundance of gut microbiota

**Figure 3:**
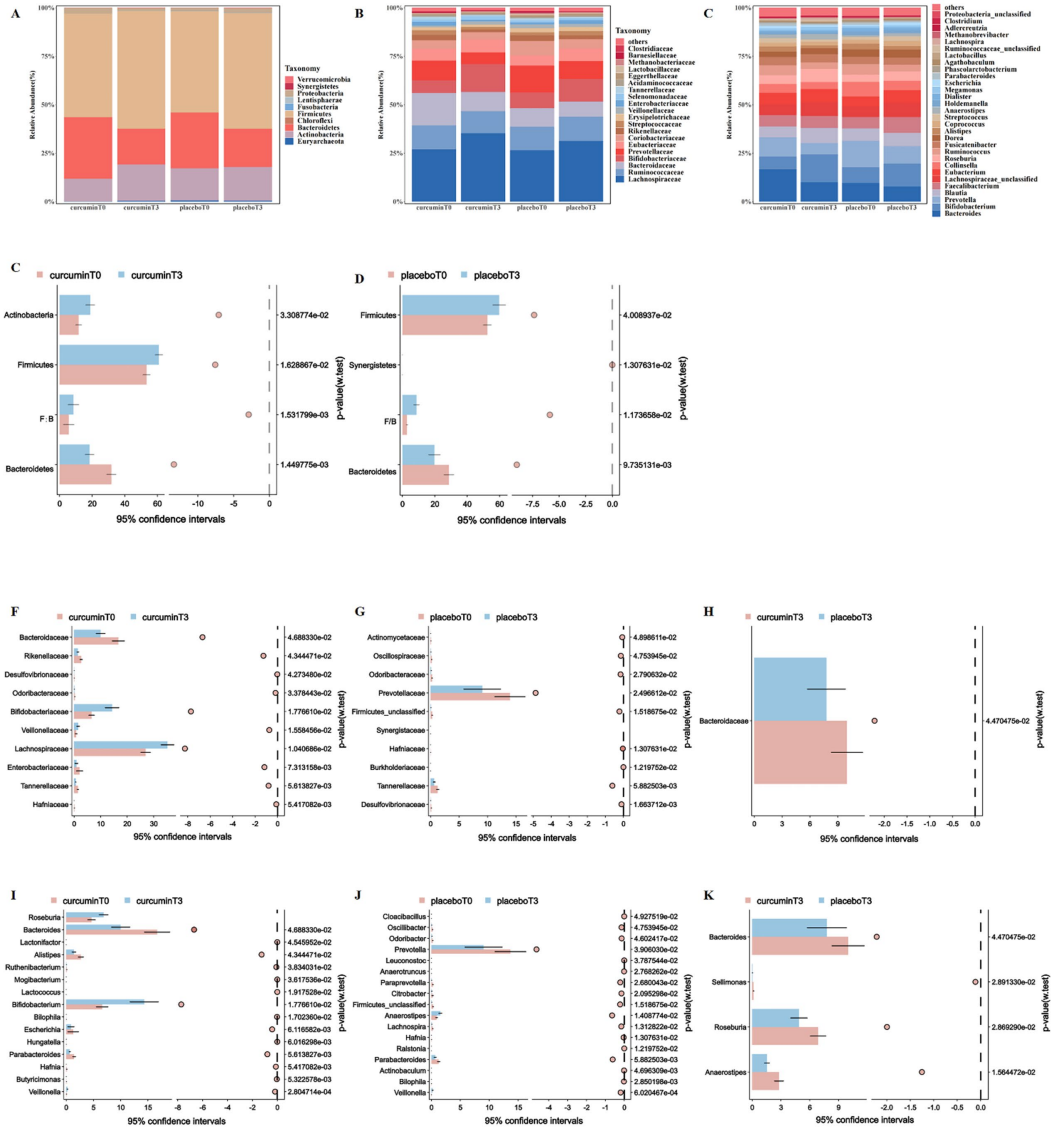
Composition of gut microbiota at the phylum **(A)**, family **(B)**, and genus **(C)** levels; and the differences in the relative abundance of gut microbiota at the phylum **(D,E)**, family **(F–H)** and genus **(I–K)** levels observed at T_0_ and T_3_ in the placebo and curcumin groups. ^*^p < 0.05, ^**^p < 0.01, and ^***^p < 0.001.

To further explore the effects of curcumin intervention on gut microbiota composition, the relative abundances of the top 10 bacteria at the phylum levels, the top 20 bacteria at the family, and the top 30 bacteria at the genus levels were compared in groups (Figures 3A–C).

At the phylum level, the relative abundance of *Firmicutes* and the ratio of *Firmicutes* and *Bacteroidetes* significantly increased, in contrast, the relative abundance of *Bacteroidetes* significantly decreased when they entered plateaus for 6 weeks in both groups (*p* < 0.05, Figures 3D,E). The relative abundance of *Actinobacteria* significantly increased after a 6-week curcumin intervention, whereas the relative abundance of *Synergistetes* significantly decreased in the placebo group (*p* < 0.05).

At the family level, the relative abundance of *Tannerellaceae* significantly decreased in both groups when they entered plateaus for 6 weeks. After curcumin intervention, the relative abundance of *Bacteroidaceae*, *Enterobacteriaceae*, and *Rikenellaceae* significantly reduced, but the relative abundance of *Bifidobacteriaceae*, *Lachnospiraceae*, and *Veillonellaceae* significantly increased (*p* < 0.05). The relative abundance of *Prevotellaceae* was significantly reduced in the placebo group after 6 weeks of plateau exposure, and the relative abundance of *Bacteroidaceae* was significantly lower than that in the curcumin group (*p* < 0.05; Figures 3F–H).

At the genus level, the relative abundance of *Anaerostipes*, *Bacteroides,* and *Parabacteroides* significantly decreased in both groups when they entered plateaus for 6 weeks, however, the relative abundance of *Veillonella* significantly increased. Participants receiving curcumin treatment from plain to plateau had a lower relative abundance of *Alistipes* and *Escherichia*, whereas they had a higher relative abundance of *Bifidobacterium* and *Roseburia* (*p* < 0.05, Figure 3I). The relative abundance of *Lachnospira*, *Odoribacter*, *Oscillibacter*, *Paraprevotella*, and *Prevotella* significantly decreased in the placebo group when they entered plateaus for 6 weeks (*p* < 0.05; Figure 3J). Meanwhile, participants in the curcumin group had a higher relative abundance of *Anaerostipes*, *Bacteroides*, *Sellimonas*, and *Roseburia* than those in the placebo group (*p* < 0.05; Figure 3K).

### 3.7 Effect of curcumin supplementation on gut microbiota biomarkers

**Figure 4:**
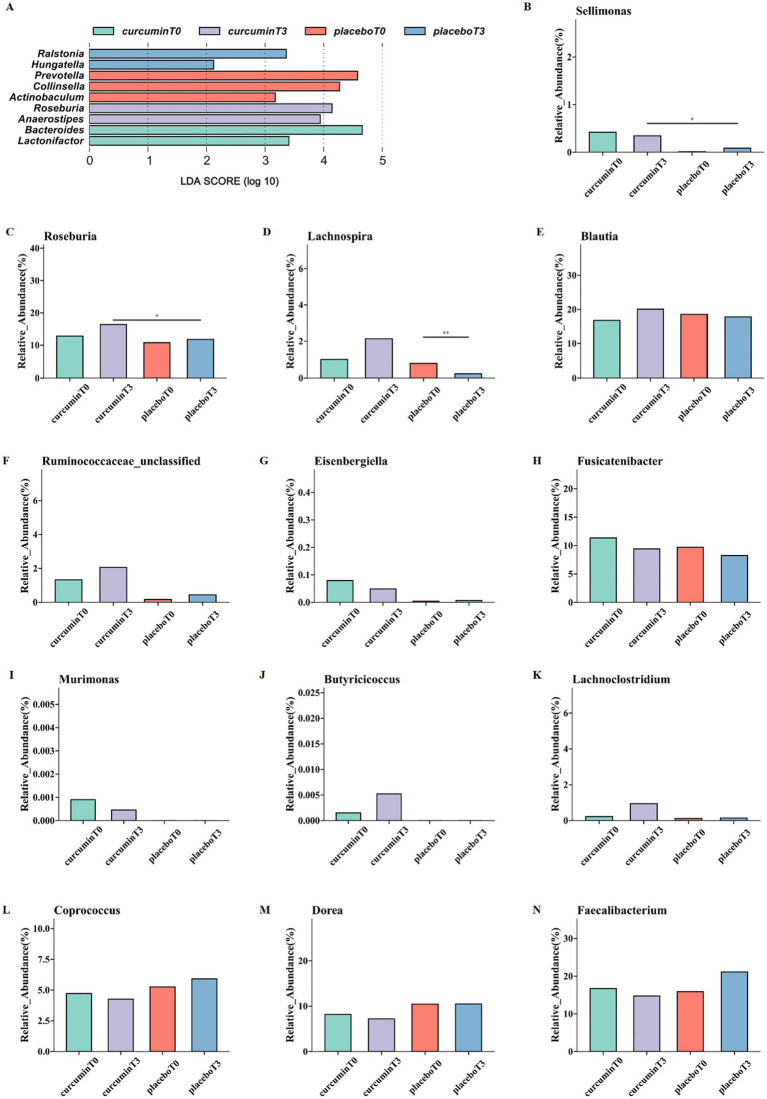
LEfSe analysis **(A)** and differences in the relative abundance of butyrate-producing bacteria at the genus levels **(B–N)**; the relative abundance of *Sellimonas* **(B)**, *Roseburia* **(C)**, *Lachnospira* **(D)**, *Blautia* **(E)**, *Ruminococcaceae* **(F)**, *Eisenbergiella* **(G)**, *Fusicatenibacter* **(H)**, *Murimonas* **(I)**, *Butyricicoccus* **(J)**, *Lachnoclostridium* **(K)**, *Coprococcus* **(L)***, Dorea* **(M)***, and Faecalibacterium* **(N)** observed at T_0_ and T_3_ in the placebo and curcumin groups. ^*^p < 0.05, ^**^p < 0.01, and ^***^p < 0.001.

The dominant bacterial communities in each group were analyzed by linear discriminant analysis effect size (LEfSe), with a total of 9 biomarkers scoring over 2 in linear discriminant analysis (LDA; Figure 4A). The superior genera of the curcumin group at T_3_ were *Roseburia* and *Anaerostipes*, while the representative genera of the curcumin group at T_0_ were *Bacteroides* and *Lactonifactor*. The gut microbiota of the placebo group at T_3_ was represented by *Ralstonia* and *Hungatella*, which was characterized by *Actinobaculum, Collinsella,* and *Prevotella* on the plain.

The relative abundance of 13 butyrate-producing bacteria was further analyzed. At the genus level, the relative abundance of *Roseburia* and *Sellimonas* significantly increased after a 6-week curcumin intervention compared to those of the placebo group (*p* < 0.05; Figures 4B,C). In addition, the relative abundance of *Lachnospira* significantly decreased in the placebo group while increased in the curcumin group (*p* < 0.05; Figure 4D). Meanwhile, the higher abundance of *Blautia, Butyricicoccus, Lachnoclostridium, Ruminococcaceae, Eisenbergiella, Fusicatenibacter, Murimonas* and lower *Coprococcus, Dorea, and Faecalibacterium* were observed in the curcumin group than those in the placebo group (Figures 4E–N).

### 3.8 Correlations between gut microbiota and high-altitude acclimatization

**Figure 5:**
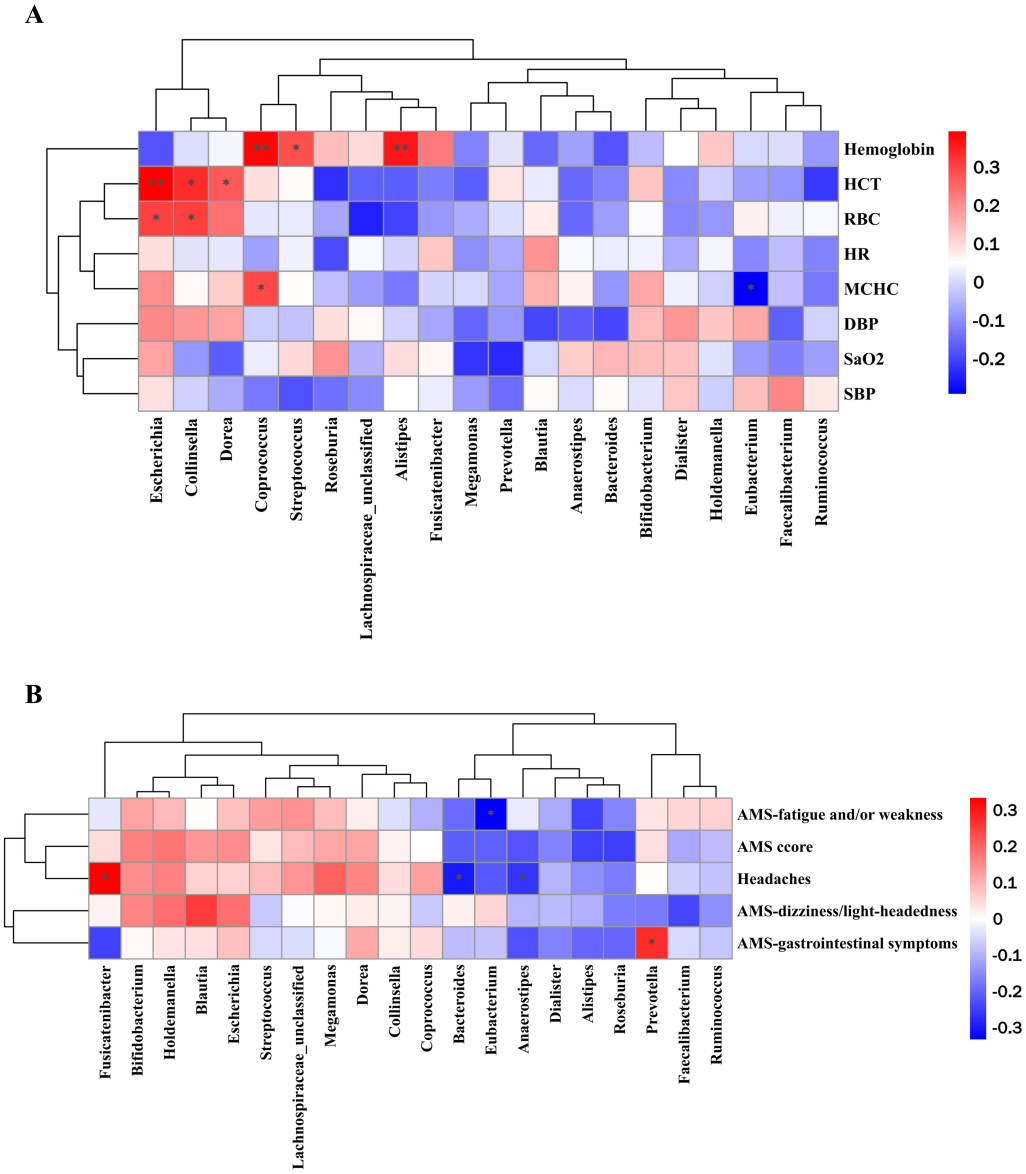
Pearson correlation analysis of top 20 bacteria at the genus level with **(A)** high-altitude acclimatization-related indicators and **(B)** AMS and AMS-related symptoms. ^*^p < 0.05, ^**^p < 0.01, and ^***^p < 0.001.

Pearson’s correlation analysis was conducted to explore the association between alterations in the relative abundance of gut microbiota and various clinical outcomes (Figures 5A,B). Notably, a significantly positive association was observed between hemoglobin and the relative abundance of *Alistipes*, *Coprococcus* (*p* < 0.01), and *Streptococcus* (*p* < 0.05). The relative abundance of *Escherichia* and *Collinsella* was significantly positively associated with HCT and RBC (*p* < 0.05, *p* < 0.01), while *Dorea* was only positive associations with HCT (*p* < 0.05). In addition, MCHC show significantly negative associations with *Eubacterium* (*p* < 0.05), but positive associations with *Coprococcus* (*p* < 0.05).

Significantly negative associations were observed between *Eubacterium* and the scores of fatigue and/or weakness. The score of headaches was positively correlated with the relative abundance of *Fusicatenibacter* while negatively correlated with *Bacteroides* and *Anaerostipes.* In addition, a significantly positive association was observed between the relative abundance of *Prevotella* and the score of gastrointestinal symptoms.

## 4 Discussion

In this randomized controlled trial conducted in 102 male Han participants urgently entered the 3,000 m plateau, we found that curcumin intake (812 mg/d) on one hand elevated the concentration of hemoglobin in subjects on the plain (1 week), on the other hand, inhibited the excessive increase of hemoglobin after entering the plateau (6 weeks). Although this trial did not uncover significant effort in improving AMS score and all hypoxia-related symptoms for curcumin supplementation, there was a trend for improving gastrointestinal symptoms of fatigue and regulating blood pressure. Due to the rise of altitude, the relative abundance of *Firmicutes* and the ratio of *Firmicutes* and *Bacteroidetes* significantly increased, whereas the relative abundance of *Bacteroidetes* significantly decreased. After curcumin consumption, a significantly higher relative abundance of probiotic bacteria *Bifidobacterium* and butyrate-producing bacteria *Roseburia*, *Lachnospira,* and *Sellimonas* was observed, while a decrease of the abundance of *Alistipes* and *Escherichia* was detected.

Unlike permanent Tibetan residents on the Qinghai-Tibet Plateau, who tend to have stable gut microbiota, the composition and diversity of the Han population’s gut microbiota may change to adapt to the high-altitude environment. Li et al. (38) compared the microbiome of the Han population living in Chengdu (500 m) and the immigrant Han population living in Lhasa (3,600 m), revealing that the latter had a more energy-efficient flora characterized by a higher ratio of *Firmicutes* to *Bacteroides*. Ma et al. (39) also revealed that high altitude may be associated with an increased ratio of *Firmicutes* to *Bacteroides* and *Firmicutes* abundance while decreased Bacteroides abundance, which was in accordance with our results. *Firmicutes* are primary SCFA-producing bacteria at the phylum. SCFAs not only improve intestinal homeostasis and protect the integrity of the intestinal epithelial barrier but also are a major energy source for epithelial cells and provide about 10–15% of daily calories in humans (40). Appropriate increases of *Firmicutes* are associated with the highly efficient extraction of energy from food to cover the increased energy requirements of high-altitude environments.

This study found that curcumin intervention increased the relative abundance of *Bifidobacterium* and *Roseburia* and decreased *Alistipes* and *Escherichia* at the genus level compared to baseline. Furthermore, participants receiving curcumin supplementation had a higher relative abundance of *Roseburia*, *Bacteroides* and *Sellimona* than those in the placebo group. *Bifidobacterium* and *Bacteroides* are commonly known as probiotics, while *Anaerostipes*, *Sellimonas,* and *Roseburia* are representative butyrate-producing bacteria. Other butyrate-producing bacteria of the top 50 genera were further analyzed and the relative abundance of *Blautia, Butyricicoccus, Lachnoclostridium, Ruminococcaceae, Eisenbergiella, Fusicatenibacter,* and *Murimonas* increased after curcumin consumption when compared to the placebo group at T_3_. Based on the above, it is reasonable to assume that curcumin facilitates butyrate-producing bacteria colonizing the gut in a high-altitude environment. Recent studies reported that a higher concentration of butyrate-producing bacteria was found in high-altitude residents than migrants, which may be one of the reasons for high-altitude adaption (41). The Mendel randomization analysis based on genome-wide association analysis data revealed that some butyrate-producing bacteria in plateau Tibetan were negatively correlated with HAPC, such as *Roseburia*, *Bilophila*, *Prevotella 9*, *Eubacterium oxidoreducens group*, *Lachnospira*, and *Ruminococcaceae UCG005* (24).

The increase in hemoglobin of this study is within normal range of human average at 3000 meters altitude. World Health Organization recommended that adjustments to be made to the measured hemoglobin concentration among populations living at an altitude of 3,000 meters (42). The adjustment reference value in our trail in the plateau should be in the range of 149–194 g/L, with the reference value from 130 to 175 g/L in the plain (19 g/L added for the altitude adjustment). Consequently, hemoglobin concentrations at 3 time-points in both groups were all in the normal reference range. A previous study revealed that average hemoglobin levels were 146.12 ± 15.5 g/L in the Han population in plain, 175.86 ± 10.4 g/L in the plateau-acclimatized Han population (residing for 30 days on the plateau), and as high as 181.98 ± 16.6 g/L in the plateau-acclimatized Han population (residing for 90 days on the plateau) (13). In this study, average hemoglobin concentration was 176.07 ± 13.78 g/L in the placebo group and 170.95 ± 8.31 g/L in the curcumin group at T_3_ (residing for 42 days on the plateau), which was comparable to other studies. Under hypoxia circumstances, the concentration of hemoglobin is modulated by hypoxia-inducible factor-1α (HIF-1α), which is a master transcriptional regulator of numerous genes important to processes that include erythropoiesis, angiogenesis, energy metabolism, and inflammation (43). Previous research suggested that excessive HIF-1α expression in hypoxic conditions at high altitudes may increase the risk of HAPC (44). However, Zhao et al. (24) performed animal experiments that demonstrated intracellular lactate accumulation in hypoxia environment, but butyrate consumption can prevent excessive accumulation of HIF-1α by inhibiting lactate dehydrogenase A activity. Therefore, we assumed that the administration of curcumin on the plateau increased butyrate in the gut and inhibited polycythemia and decreased hemoglobin to prevent HAPC.

Another mechanism of curcumin that prevents HAPC may relate to enhancing oxygen supply efficiency and improving adaption to oxygen-deficient environments. Polycythemia is more prevalent among the Han population living on the plateau than among Tibetan residents, which is regarded as a blunted physiological response at high altitudes to protect Tibetans from erythrocytosis (45). The average hemoglobin levels of the plateau Tibetan population at an altitude of about 5,000 m were 170 g/L for men and 140 g/L for women, which are significantly lower than those of Han populations who urgently entered the plateau at the same altitude (46). Webb et al. (14) first proposed that high oxygen affinity hemoglobin can increase arterial oxygen saturation and alleviate hypoxia-induced accelerated heart rate without causing hemoglobin increase in the plateau hypoxic environment. Regulation of hemoglobin oxygen affinity is an important physiological regulation of the organism in response to acute hypoxia (47). Therefore, both the quantity and functional quality of RBC and hemoglobin are equally important. Chou’s team found that curcumin could bind to the heme region on the *α*-subunit of hemoglobin to increase the structural stability of hemoglobin, decrease the partial pressure of oxygen at half saturation (P50), enhance the Bore effect, and improve oxygen-carrying capacity by *in vitro* studies. Subsequently, they conducted animal experiments to verify the regulatory effects of curcumin *in vivo* (29). Their results showed that both 50 mg/kg and 100 mg/kg curcumin administration significantly prolonged the survival time of asphyxia tolerance and enhanced the hypoxia tolerance ability of the organism under extreme hypoxic conditions in mice. In addition, daily administration of 200 mg/kg curcumin to rats with acute plateau hypoxia at a simulated altitude of 6,000 m lowered the P50 by 20.03% and the number of erythrocytes by 18.07%, increased the acid–base sensitivity index by 23.38% and the theoretical oxygen-releasing capacity by 43.72%, as well as alleviated the damage to the organs. The above results suggest that curcumin can enhance hemoglobin–oxygen affinity instead of increasing the counts of RBC and hemoglobin to promote high-altitude acclimatization. Unfortunately, there is insufficient evidence to support the role of gut flora in this process.

Interestingly, the hemoglobin concentration of the curcumin group (160.18 ± 1.35 g/L) was significantly higher than that of the placebo group (155.57 ± 1.32 g/L) after 1 week of intervention on the plain. Hemoglobin levels increased in the first week of intervention at the plain in both groups, which was significantly higher in the curcumin group than in the placebo group. The early increase in hemoglobin concentration in both groups may be attributed to both the routine physical activity habit and the intervention. Sedliak (48) revealed that a 6-month army drill significantly increased the concentration of hemoglobin and hematocrit in all subjects whose baseline clinical physiological values were all normal. Meanwhile, previous evidence suggested that curcumin consumption in humans was potentially safe and beneficial for exercise and physical activity (49–51), which might account for the higher hemoglobin levels of the curcumin group on the plain.

In this study, a significant decrease in SBP on the plain in the curcumin group was observed when compared to the placebo group. The level of DBP increased at altitudes of 3,000 m in the curcumin group which was still significantly lower than that in the placebo group. According to previous studies, blood pressure is persistently elevated at altitudes between 3,000 and 5,000 m, and continues to increase during the period of exposure (52, 53). Moreover, the changes in DBP were more pronounced relative to SBP because of the greater effect of hypoxia on arterial elasticity (54). To the best of our knowledge, current research on the effects of curcumin on SBP and DBP is mostly focused on patients with obesity, diabetes mellitus, and metabolic syndrome, without literature accessing the effects of curcumin on blood pressure in healthy adults on both plain and high altitudes. Karimi (55) reported that curcumin did not improve SBP between intervention and placebo groups, but there was a significant reduction in DBP after curcumin administration over 12 weeks in the systematic review. These inconsistent results can be due to different doses and formulations of curcumin, different baseline of blood pressure, altitude, and lack of large-scale data (56, 57). Blood pressure can be also related to gut microbiota in high-altitude populations of humans, for example, which have reported that olfactory receptor 78 in the kidney can increase blood pressure through SCFA-mediated renin release (58, 59). In this study, no direct correlation between gut microbiota and blood pressure was found.

Based on the data released by the Center for Alpine Disease Prevention and Control from the General Hospital of Tibet Military Region, the incidence of AMS in the plateau at an altitude of 3,000 m was 56.47% (60), which was significantly higher than the incidence reported in our study. It was attributed to the fact that the subjects in this study were all healthy male Han personnel, all of whom had passed health checkups before entering the plateau, and had regular physical fitness and adaptability. After 6 weeks into the plateau, the incidence of AMS in the curcumin group and placebo group increased. We further analyzed the possible reasons and concluded that the study was conducted in winter with a higher incidence of AMS than that in other seasons. Notably, the scores of gastrointestinal symptoms, fatigue and/or weakness, and AMS in the curcumin group were lower than those in the placebo group at T_3_, though the differences were not significant. Our findings were in good agreement with prior studies. A randomized, double-blind, placebo-controlled study showed that daily consumption of the 500 mg curcumin extract for 8 weeks significantly relieved gastrointestinal symptoms in adults with self-reported digestive complaints (61). It has been hypothesized that curcumin not only plays a role in the gastrointestinal tract directly but also interacts with gut microbiota to exert health effects indirectly (62).

To our knowledge, this might be the first randomized clinical trial to determine the efficacy of curcumin on high-altitude acclimation in the Han population, but there are several limitations. Firstly, the study population consisted of young males with an average age of 20 years, whose physiological metabolic rates, hormone levels and baseline hemoglobin values may be significantly different from those of other age groups or female groups. High altitude adaptation is associated with genetic polymorphisms, such that both plateau-dwelling Tibetans and Chinese Han population are more adaptable to high altitude than plain-dwelling Chinese Han population. However, the present study focused only on the plains-dwelling Chinese Han population. Further research is needed if the findings are to be generalized to others. Secondly, the blood concentration of the curcumin or its metabolite were not detected. Last but not least, the one-year follow-up of all subjects was incomplete, due to the loss of contact; thirdly, metabolites of intestinal flora and the affinity of oxygen in hemoglobin under different altitudes were not detected. In the future, the bioavailability of curcumin *in vivo* and its molecular mechanism *in vitro* can be further depicted to provide more precise evidence for curcumin food preparation, AMS alleviation, and HAPC prevention.

## 5 Conclusion

Curcumin exhibited different regulation mechanisms of hemoglobin in low- and high-altitude environments, which may be associated with changes in gut microbiota. On the plain, curcumin supplementation elevated the concentration of hemoglobin, which is favorable to increasing the organic oxygen supply and relieving symptoms from hypoxia at the early stage of entering the plateau. On the plateau, curcumin suppressed excessive hemoglobin by modulating the abundance of butyrate-producing bacteria to prevent HAPC.

## Data Availability

The original contributions presented in the study are included in the article/Supplementary material, further inquiries can be directed to the corresponding authors.
