# Supplementary material for: Curcumin supplementation accelerates high-altitude acclimatization, prevents polycythemia and modulates gut microbiota in male Han population: a randomized controlled trial
Source: Front Nutr. 2025 Jul 8;12:1572376. doi: 10.3389/fnut.2025.1572376 (PMC12279741; doi:10.3389/fnut.2025.1572376)
Supplement: Supplementary file 1 [file Data_Sheet_1.pdf]

Table1 Chromatographic Analysis Raw Record of Curcumin Gummies

**Sample Mass (g)**	**Extract Volume (mL)**	**Dilution Factor**	**Analyte Detected**	**Concentration of Test Solution (μg/mL)**	**Result (%)**	**Average (%)**	**Relative Standard Deviation (%)**
Parallel 1: 2.0385<br>Parallel 2: 2.0349<br>Parallel 3: 2.1255	100.00	1	Curcumin	Parallel 1: 209.72<br>Parallel 2: 210.40<br>Parallel 3: 218.15	1.03<br>1.03<br>1.03	1.03	0
			Demethoxycurcumin	Parallel 1: 25.26<br>Parallel 2: 25.33<br>Parallel 3: 26.27	0.12<br>0.12<br>0.12	0.12	0
			Bisdemethoxycurcumin	Parallel 1: 1.64<br>Parallel 2: 1.64<br>Parallel 3: 1.67	0.01<br>0.01<br>0.01	0.01	0
			Total Curcumin	/	/	1.16	/

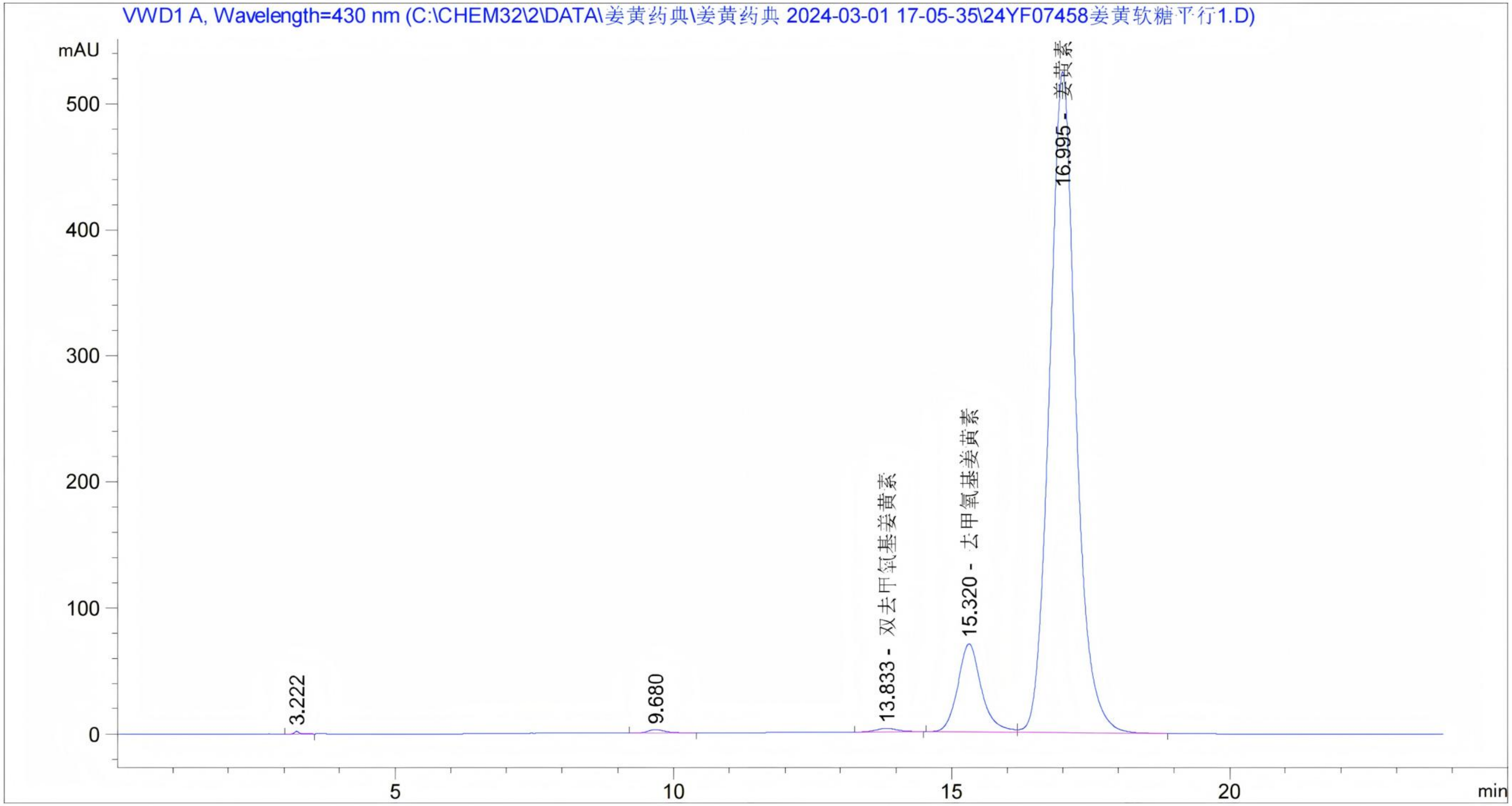

Figure 1 Chromatogram of Curcumin Gummies
